# Supplementary material for: Suppression of bacterial cell death underlies the antagonistic interaction between ciprofloxacin and tetracycline
Source: Mol Syst Biol. 2025 Oct 27;22(1):7. doi: 10.1038/s44320-025-00162-w (PMC12759072; doi:10.1038/s44320-025-00162-w)
Supplement: Supplementary file 3 — Appendix [file 44320_2025_162_MOESM3_ESM.pdf]

Appendix for "Suppression of bacterial cell death underlies the antagonistic interaction between ciprofloxacin and tetracycline"

James Broughton[1], Achille Fraisse[1], and Meriem El Karoui[1, 2*]

[1]*Institute of Cell Biology, School of Biological Sciences, University of Edinburgh, UK*

[2]*LBPA, Ecole Normale Supérieure- Paris-Saclay, CNRS UMR 8113, Gif-sur-Yvette, FRANCE*

[*]*Corresponding author: Meriem El Karoui, meriem.elkaroui@ed.ac.uk*

Contents

1 Appendix Figures and Tables **3**

Appendix Figure S1 3

Appendix Figure S2 3

Appendix Figure S3 4

Appendix Table S1 4

Appendix Figure S4 5

Appendix Figure S5 6

Appendix Figure S6 7

Appendix Figure S7 8

Appendix Figure S8 9

Appendix Figure S9 10

Appendix Figure S10 10

Appendix Figure S11 11

Appendix Figure S12 12

Appendix Figure S13 13

2 Appendix Methods **14**

2.1 List of strains, plasmids, and primers 14

Appendix Table S2: List of strains. 14

Appendix Table S3: List of plasmids. 14

Appendix Table S4: List of primers. 14

2.2 Bulk experiments 15

2.2.1 Checkerboard assay 15

2.2.2 Bulk growth rates 16

2.3 Mother machine 17

2.3.1 Microfluidic device dimensions 17

Appendix Table S5 17

2.3.2 Classification of cell fate 17

2.3.3 Quantification of cell survival and death 17

2.3.4 Classification of dead lineages into low-SOS and high-SOS inducers 18

Appendix Table S6 18

2.4 Statistical analysis of Bliss independence 19

1 Appendix Figures and Tables

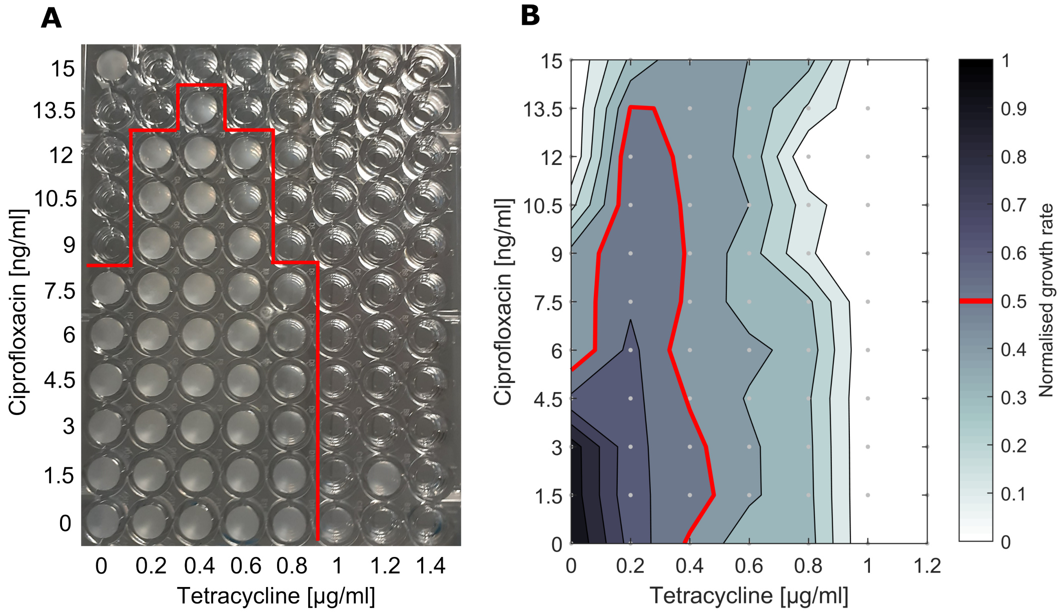

Appendix Figure S1: **Checkerboard assay showing bulk-level suppressive interaction between ciprofloxacin and tetracycline**.

A. Picture of the 96-well plate indicating growth (turbidity) in wells after 28 hours incubation. Red line indicates wells with significant growth. Wells with significant growth outside this line are likely resistant mutants and were excluded from analysis. Cells were cultured in the Glu-aa medium.

B. Dose-response surface showing growth rates (blue colour) over a two-dimensional grid of antibiotic concentrations (grey dots represent concentrations used). Colour gradient represents normalised growth rate where dark blue represents no growth inhibition and white represents no growth. Red line follows the IC_{50} isobole on the dose-response surface. The bulging convex isobole is indicative of a suppressive drug interaction according to the Loewe additivity model (Loewe, 1928, 1953). Strain SJR206 was used for the checkerboard assay. Detailed description of the protocol can be found in the Appendix Methods.

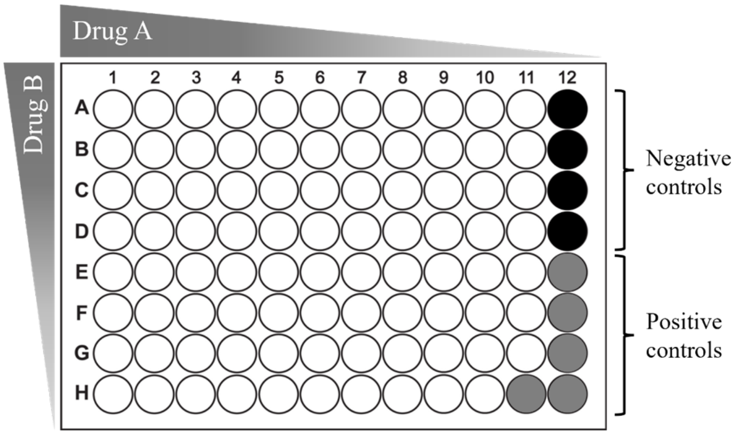

Appendix Figure S2: **Depiction of checkerboard assay in a 96-well plate used to evaluate interactions for antibiotic combinations**. A 2D concentration gradient is set up for drug A and drug B along the X and Y axis. Single drug concentration gradients are set up in Row H and Column 11 for drug A and B, respectively. The positions of the negative (black wells) and positive controls (grey wells) are indicated.

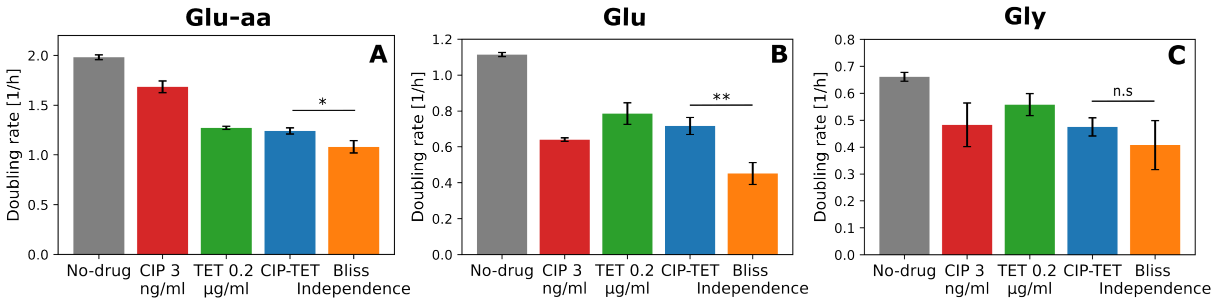

Appendix Figure S3: **Bulk doubling rates under 3 ng/mL ciprofloxacin and 0.2 μg/mL tetracycline mono-exposure and under combination treatment in three different growth media**. Shown is the mean and standard error from three biological replicates for growth in the (**A**) Glu-aa, (**B**) Glu and (**C**) Gly medium. Doubling rates were measured using automatic OD_{600} measurements from OGI-BIO bioreactors for strain JKB43 (Appendix Table S1). See Appendix Methods for the detailed experimental protocol. Similar to the survival fractions, the Bliss expectation was calculated using the log-transformed pairwise sums of the replicate normalised doubling rates (λ) in each group: $Bliss_{ij} = \exp(\ln(\frac{\lambda_{CIP_{ij}}}{\bar{\lambda}_{0_i}}) + \ln(\frac{\lambda_{TET_{ij}}}{\bar{\lambda}_{0_i}}))$, where i=1,2,3 for the three growth media, j=1,2,3 for the three biological replicates in the ith group, and $\bar{\lambda}_{0_i}$ is the mean doubling rate of the no-drug control in the ith group (Demidenko & Miller, 2019). Finally, $Bliss_{ij}$ was multiplied by $\bar{\lambda}_{0_i}$ to get the Bliss additive prediction. Error bars and statistical analysis were calculated as done previously for the survival fractions (see Methods). Significant differences are indicated: $**p<0.005$, $*p<0.05$, n.s.=not significant (one-sided t-test, ANOVA model described in Methods). Since the observed doubling rate under CIP-TET are significantly higher than the Bliss expectation in the Glu-aa and Glu growth medium, this indicates an antagonistic drug interaction in these conditions.

Appendix Table S1: **Bulk doubling rates and doubling times under ciprofloxacin and tetracycline mono-exposure and under combination treatment in three different growth media**. Doubling rates were measured using automatic OD_{600} measurements from OGI-BIO bioreactors for strain JKB43. Three biological replicates were performed for each condition. Bliss independence was calculated as described in Appendix Figure S3. S.E.M = standard error of the mean.

	**Mean doubling rate [1/h]**					**S.E.M [1/h]**				
	Control	CIP	TET	CIP-TET	Bliss	Control	CIP	TET	CIP-TET	Bliss
Glu-aa	1.981	1.684	1.272	1.241	1.081	0.024	0.059	0.017	0.031	0.062
Glu	1.114	0.641	0.786	0.716	0.452	0.011	0.010	0.060	0.047	0.061
Gly	0.661	0.483	0.558	0.475	0.407	0.016	0.081	0.041	0.034	0.091

	**Mean doubling time [min]**				**Standard deviation [min]**			
	Control	CIP	TET	CIP-TET	Control	CIP	TET	CIP-TET
Glu-aa	30.34	36.00	47.21	48.49	1.560	4.397	1.640	3.151
Glu	53.86	93.69	77.19	84.32	1.304	1.900	9.586	8.267
Gly	90.84	126.79	108.13	126.75	3.180	21.63	9.184	9.172

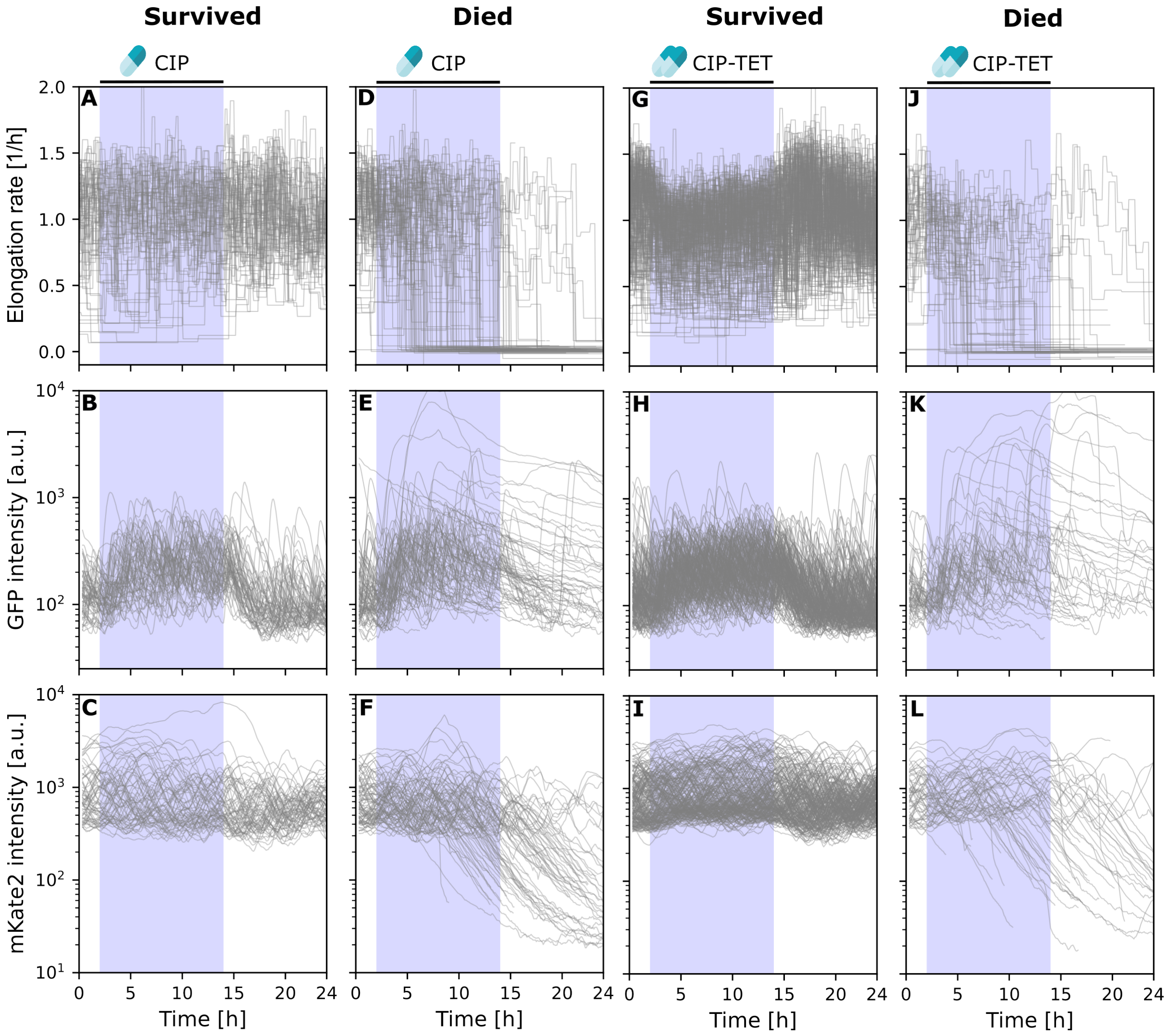

Appendix Figure S4: **Single-cell responses separated by cell fate for the Glu-aa growth medium**. Lineage fate at the end of the experiment was classified as 'survived' or 'died' as described previously.

A-F. Single-cell elongation rates, SOS expression, and constitutive expression under CIP treatment.

G-L. Single-cell elongation rates, SOS expression, and constitutive expression under CIP-TET treatment.

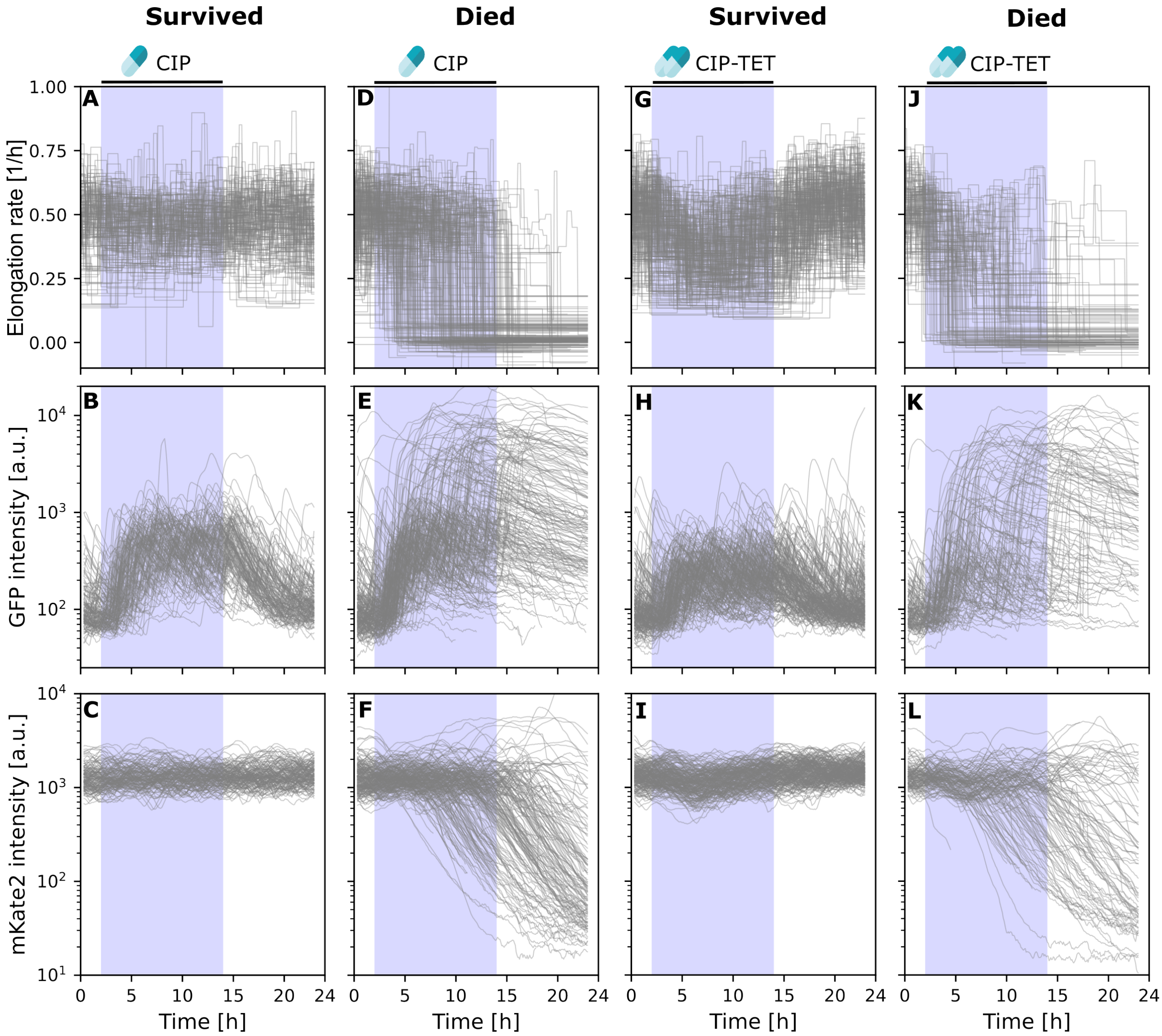

Appendix Figure S5: **Single-cell responses separated by cell fate for the Glu growth medium**. Lineage fate at the end of the experiment was classified as ‘survived’ or ‘died’ as described previously. **A-F**. Single-cell elongation rates, SOS expression, and constitutive expression under CIP treatment. **G-L**. Single-cell elongation rates, SOS expression, and constitutive expression under CIP-TET treatment.

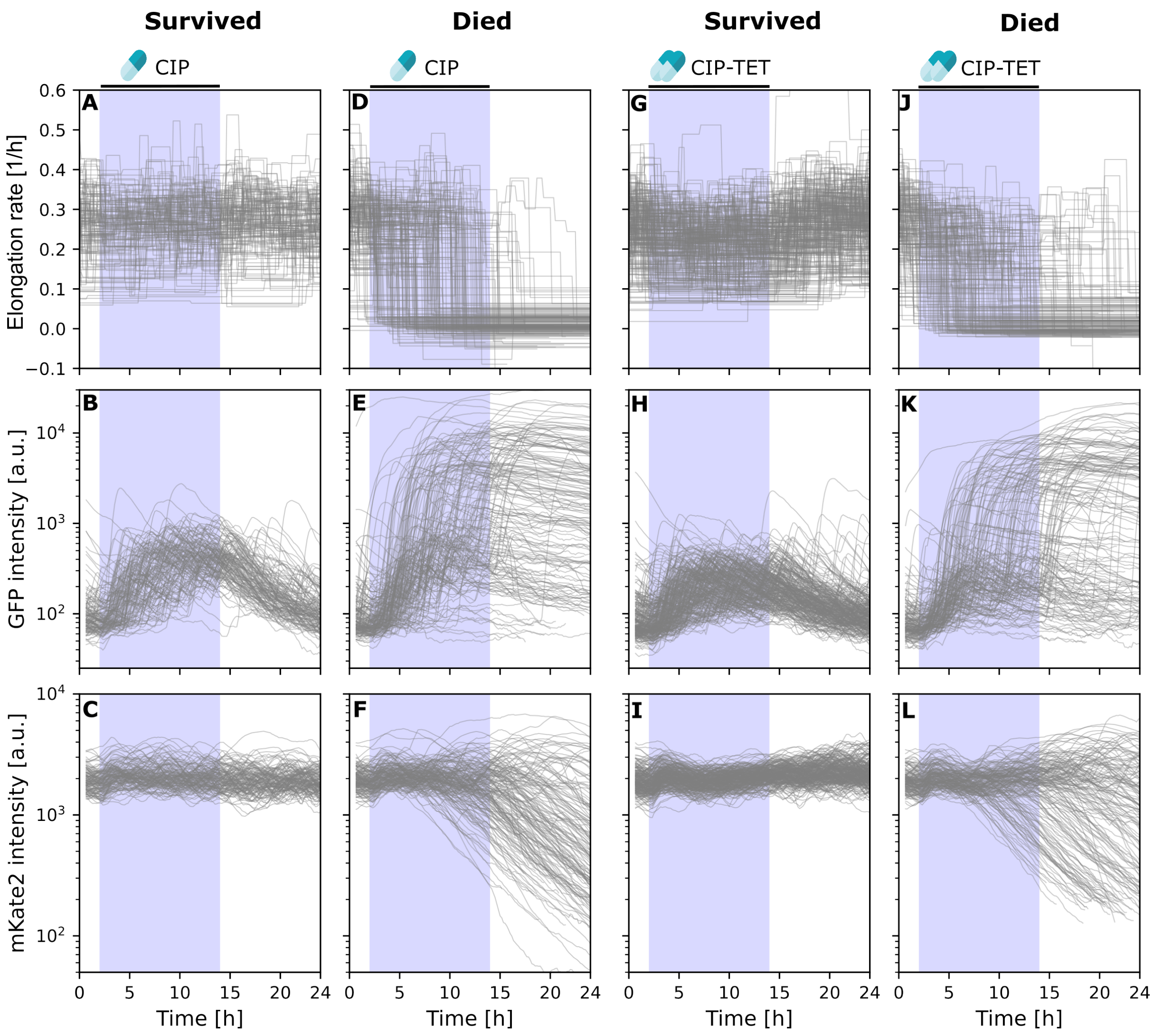

Appendix Figure S6: **Single-cell responses separated by cell fate for the Gly growth medium**. Lineage fate at the end of the experiment was classified as ‘survived’ or ‘died’ as described previously. **A-F**. Single-cell elongation rates, SOS expression, and constitutive expression under CIP treatment. **G-L**. Single-cell elongation rates, SOS expression, and constitutive expression under CIP-TET treatment.

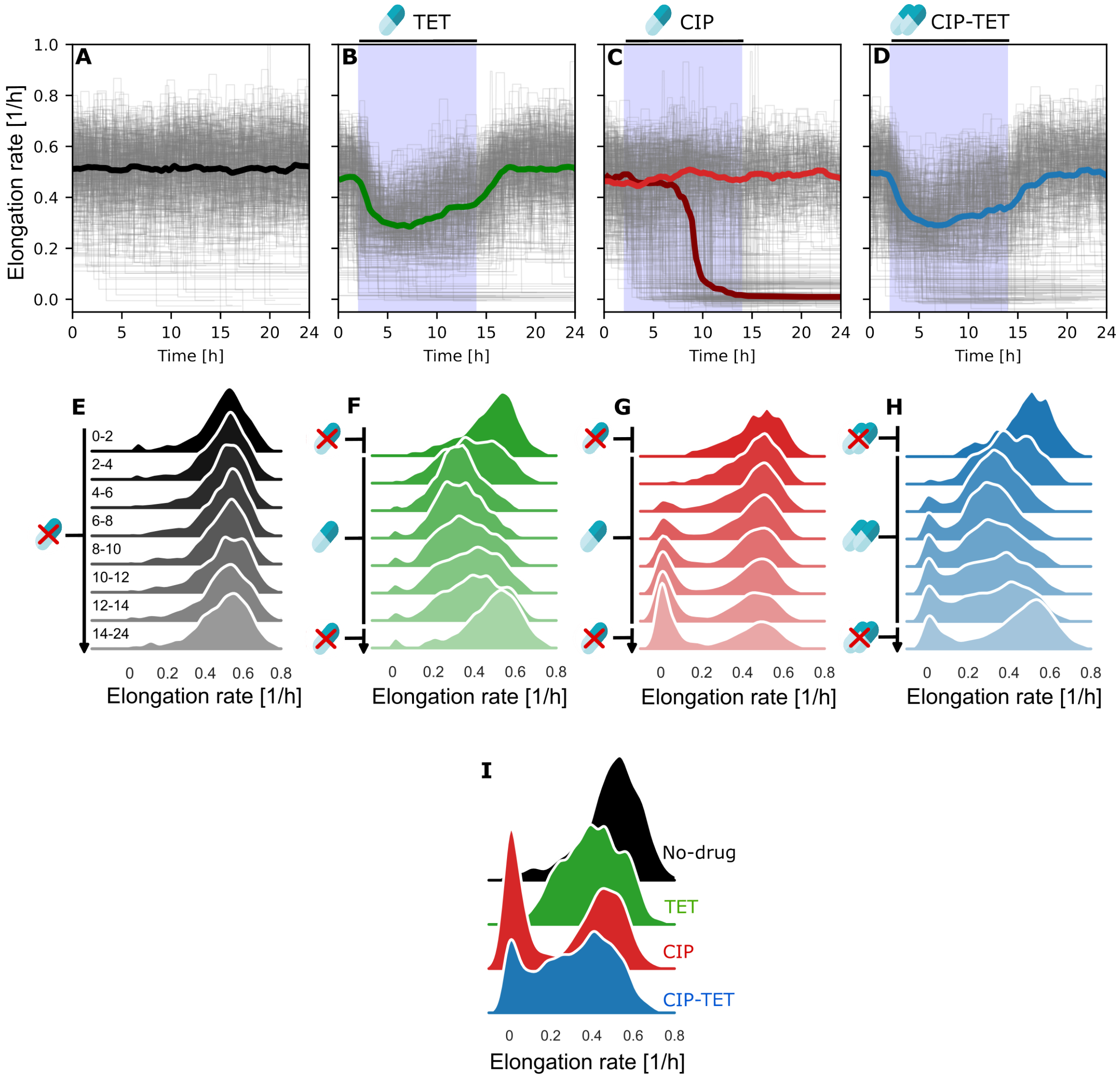

Appendix Figure S7: **Single-cell elongation rates under sub-lethal antibiotic treatment for growth in the Glu medium.**

A-D. Single-cell trajectories of elongation rates under no-drug (A, n=342 cells), 0.2 μg/mL tetracycline (B, n=351), 3 ng/mL ciprofloxacin (C, n=386), and CIP-TET (D, n=355) treatment. Antibiotics were introduced between hours 2-14, indicated by the blue shaded area. Grey lines represent individual mother cell lineage trajectories. Solid lines represent the median elongation rate of the population. In C, the red solid line represents cells that survived CIP treatment and the maroon solid line represents cells that stopped growth under CIP treatment. Shown is data from one experimental replicate for growth in the Glu medium.

E-H. Distributions of single-cell elongation rates for the no-drug control (E), TET treatment (F), CIP treatment (G), and CIP-TET treatment (H) drawn from sequential two-hour periods under different antibiotic treatments. Distributions are kernel density estimates of the underlying histogram pooled from at least two experimental replicates. Time periods, in hours, are indicated to the left of each distribution. Antibiotics were introduced from hours 2-14 as indicated.

I. Distributions of single-cell elongation rates drawn from the final 2 hours of antibiotic treatment.

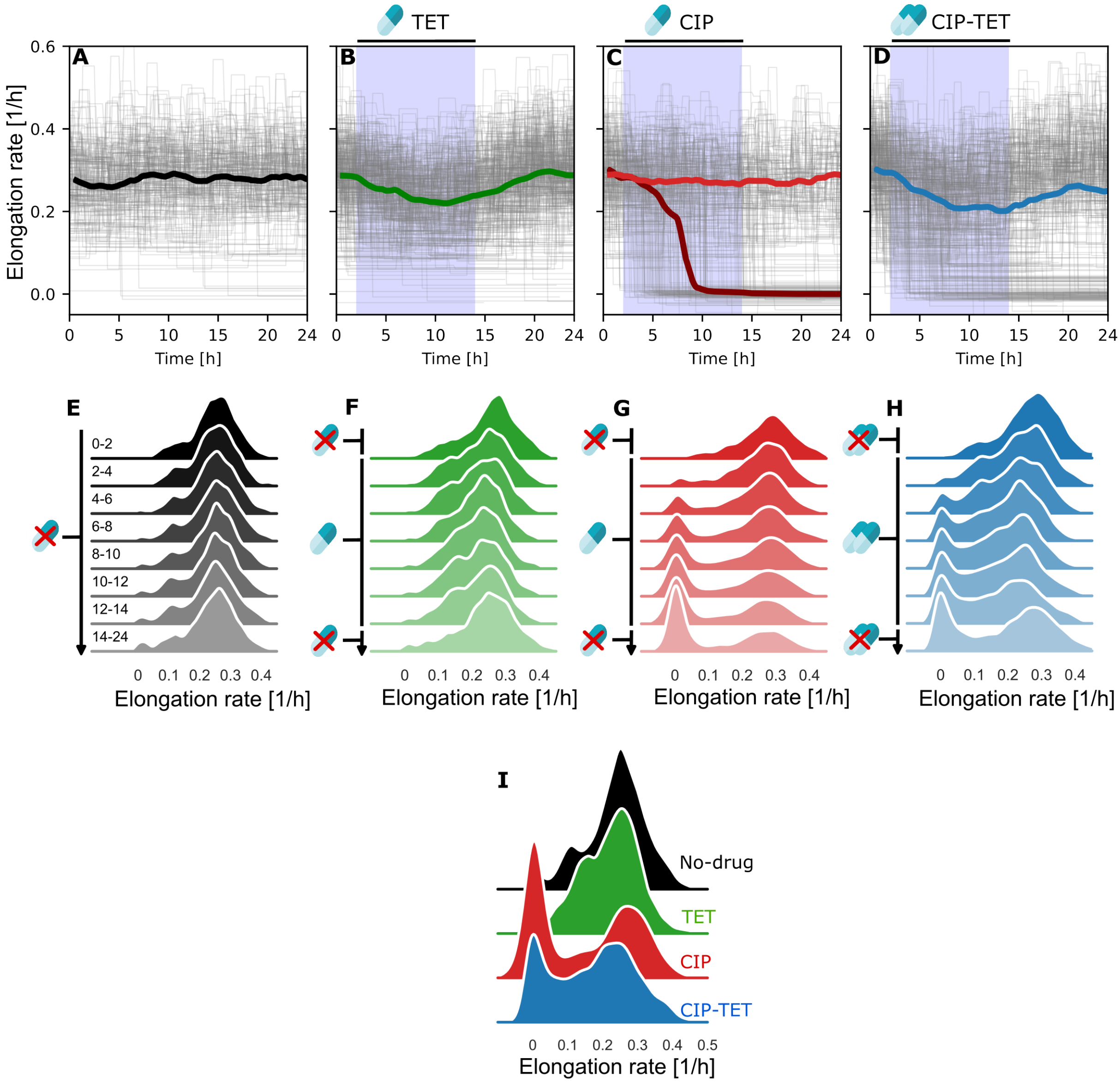

Appendix Figure S8: **Single-cell elongation rates under sub-lethal antibiotic treatment for growth in the Gly medium.**

A-D. Single-cell trajectories of elongation rates under no-drug (A, n=188 cells), 0.2 μg/mL tetracycline (B, n=317), 3 ng/mL ciprofloxacin (C, n=252), and CIP-TET (D, n=328) treatment. Antibiotics were introduced between hours 2-14, indicated by the blue shaded area. Grey lines represent individual mother cell lineage trajectories. Solid lines represent the median elongation rate of the population. In C, the red solid line represents cells that survived CIP treatment and the maroon solid line represents cells that stopped growth under CIP treatment. Shown is data from one experimental replicate for growth in the Gly medium.

E-H. Distributions of single-cell elongation rates for the no-drug control (E), TET treatment (F), CIP treatment (G), and CIP-TET treatment (H) drawn from sequential two-hour periods under different antibiotic treatments. Distributions are kernel density estimates of the underlying histogram pooled from at least two experimental replicates. Time periods, in hours, are indicated to the left of each distribution. Antibiotics were introduced from hours 2-14 as indicated.

I. Distributions of single-cell elongation rates drawn from the final 2 hours of antibiotic treatment.

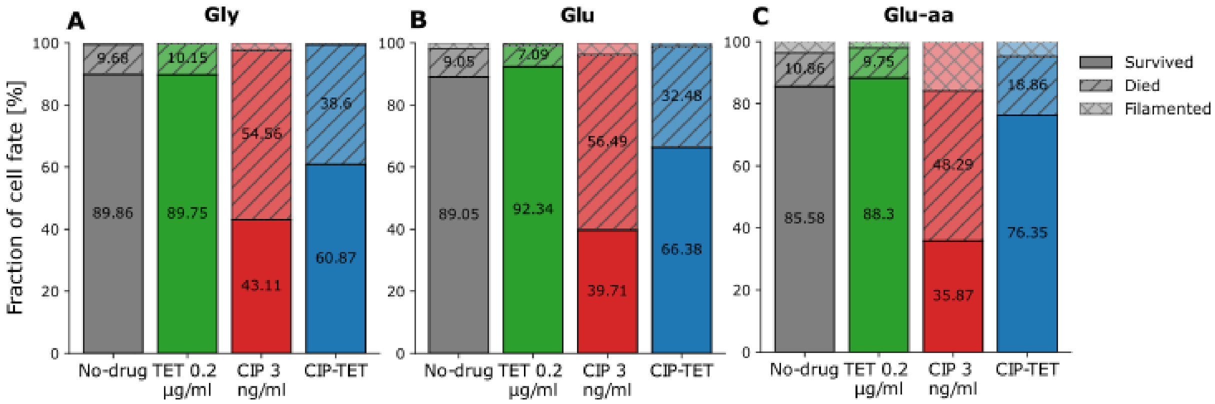

Appendix Figure S9: **Classification of lineage fate under different treatment and growth conditions**.
A-C. Shown is the fraction of cells that either survived, died (growth and division arrest or lysis), or filamented over the duration of the experiment. The fractions shown represent fates pooled from least two experimental replicates. Suppression of cell death under the CIP-TET combination is growth rate-dependent. Cell filamentation is more prevalent in fast-growth condition (Glu-aa). See Appendix Methods for detailed description of fate classification.

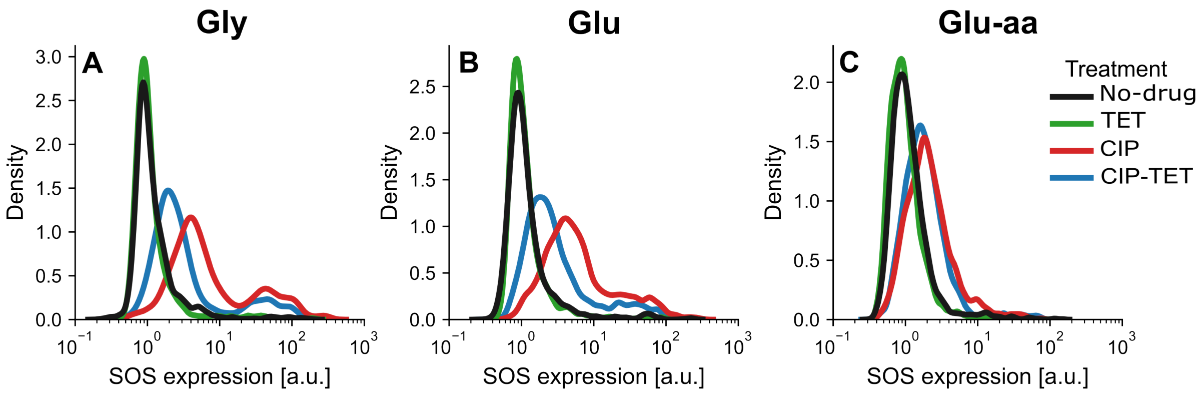

Appendix Figure S10: **Distributions of single-cell SOS expression from P_{sulA}-$mGFP$ normalised to the median of the no-drug control under TET, CIP and CIP-TET treatment and for growth in Gly (A), Glu (B) and Glu-aa (C) medium**. Distributions are kernel density estimates of the underlying histogram drawn from the final 2 hours of the antibiotic treatment period and pooled from at least two experimental replicates. This figure represents the same data shown in Figure 4 but presented in an alternative format.

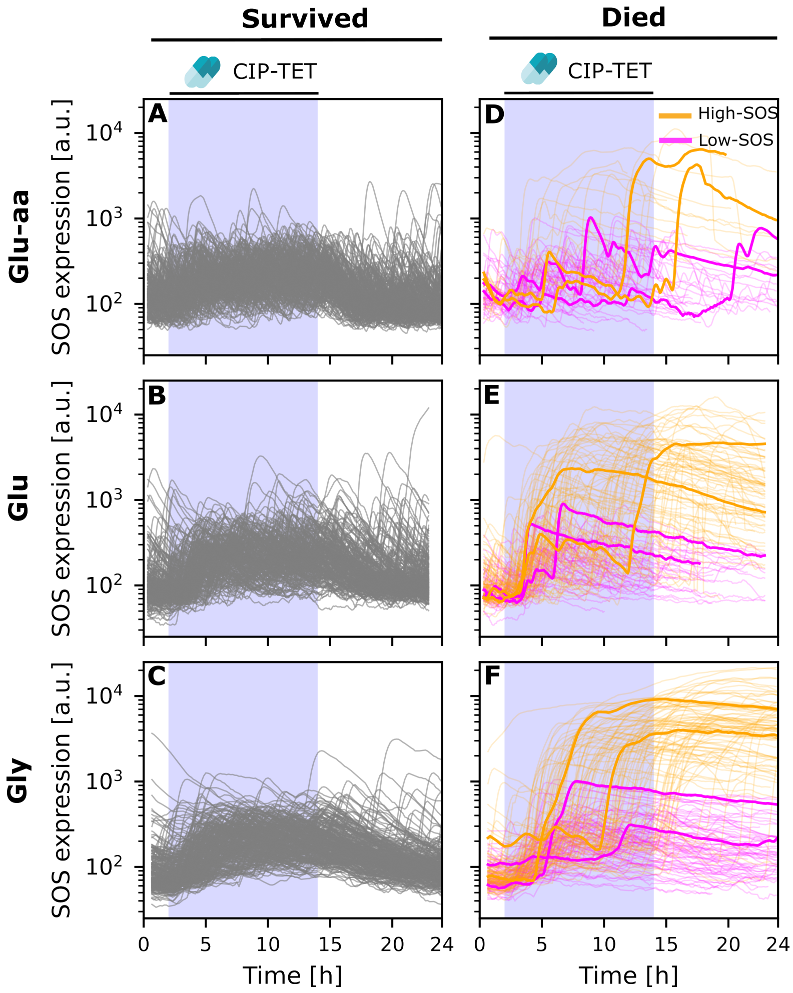

Appendix Figure S11: **The SOS response is highly heterogeneous under CIP-TET treatment**. Single-cell trajectories of SOS expression classified by cell fate (A-C: cells that survived drug treatment, D-F: cells that died) under CIP-TET treatment in different growth conditions (A,D: Glu-aa; B,E: Glu; C,F: Gly). Dead cells were further classified by expression level (low-SOS: magenta, high-SOS: orange) as described in the Appendix Methods. Example trajectories are highlighted to illustrate the behaviour of the two sub-populations. Shown is data from one experimental replicate for clarity. CIP-TET was introduced between hours 2-14 (blue shaded area). Figure panels A-C were reused from Appendix Figures S4H, S5H and S6H, respectively, in order to better visualise SOS expression trajectories between lineages that had survived and died. A: n=229 cells; B: n=220; C: n=260; D: n=58; E: n=126; F: n=168.

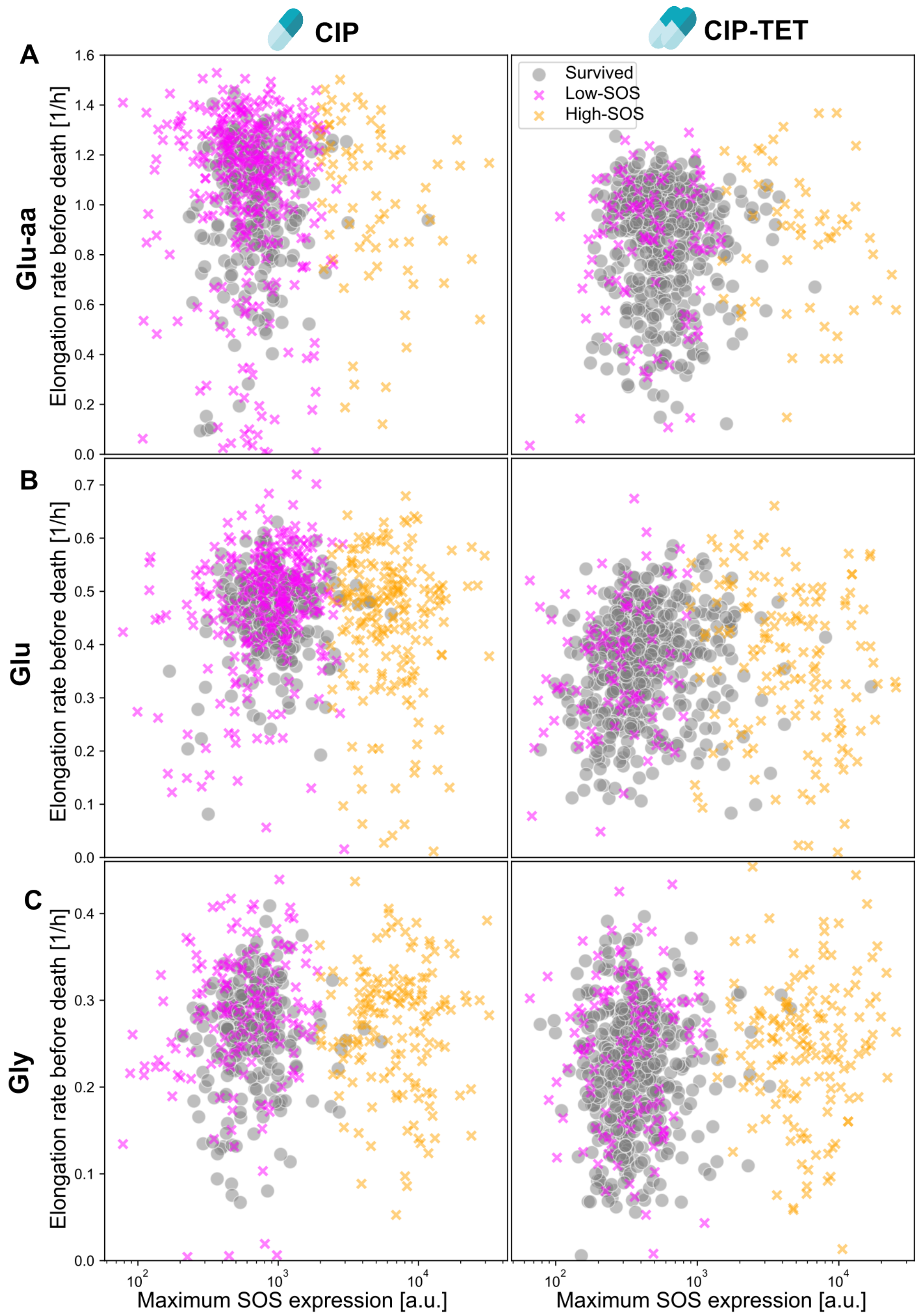

Appendix Figure S12: **Low-SOS cells are protected by treatment under the combination of antibiotics to a greater extent than high-SOS cells**. Data plotted for cells classified as low and high-SOS (magenta and orange crosses, respectively) represent lineages that died under CIP or CIP-TET treatment in Glu-aa (row A), Glu (row B), and Gly (row C) medium. The average elongation rate in the two hours preceding death for dead cells and in the second half of the antibiotic exposure period for survivors (grey circles) is plotted against the maximum SOS intensity over the whole trajectory.

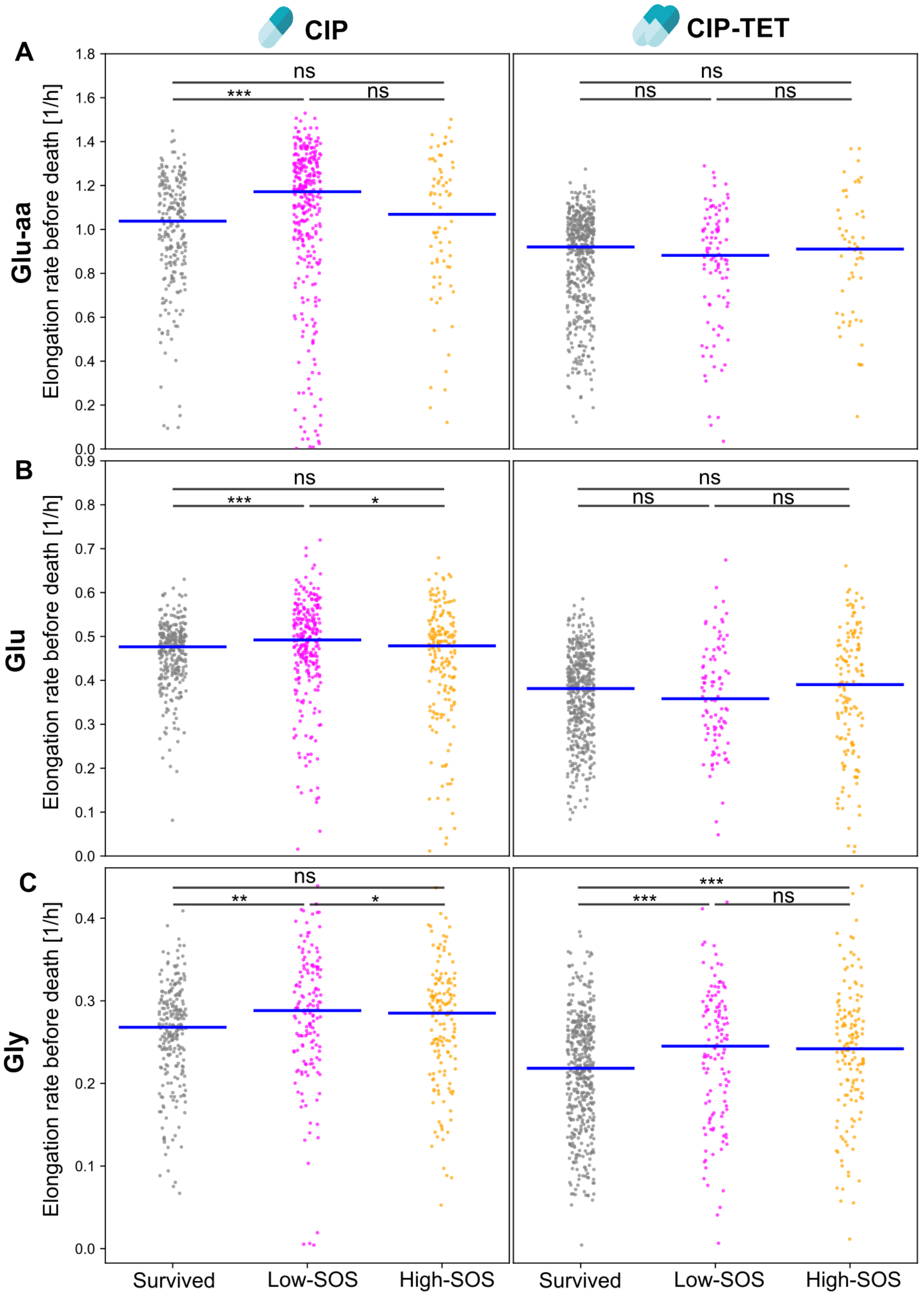

Appendix Figure S13: **Low-SOS dying cells grow faster during the two hours before they die in the CIP experiment**. Data points represent the average of single-cell elongation rates in the two hours preceding death for dead cells classified as low-SOS and high-SOS (represented by magenta and orange circles, respectively) in Glu-aa (row A), Glu (row B), and Gly (row C) medium. For surviving cells (grey circles), we show the average of single-cell elongation rates over the second half of the antibiotic exposure period which represents a steady-state response. The median of the population is indicated by the blue bar. Results of Mann-Whitney tests are indicated (ns: non significant, *: $p < 0.05$, **: $p < 0.01$, ***: $p < 0.001$.)

2 Appendix Methods

2.1 List of strains, plasmids, and primers

Here are the list of strains, plasmids, and primers used in this study. Details for the construction of the parental strain eSJR206 can be found in Jaramillo-Riveri *et al.* (2022). Bacterial strains were constructed by P1 transduction and the resistance markers removed using the pE-FLP plasmid (St-Pierre *et al.*, 2013). After construction, all strains were checked by PCR amplification and Sanger sequencing of the modified chromosomal region. The *motA* gene was deleted to reduce motility and improve retention of cells in the mother machine microchannels.

Appendix Table S2: **List of strains.** P1 stands for P1 phage transduction.

**Strain**	**Background**	**Genotype**	**Source/Construction**
JW1879-2	BW25113	*rph-1* λ^- *F- hsdR514* Δ*(araD-araB)567* Δ*(rhaD-rhaB)568* Δ*lacZ4787(::rrnB-3)* Δ*motA743::Kn*^{R}	Gift from Rosalind Allen (CGSC 9565) (Baba *et al.*, 2006)
eSJR206	MG1655	*rph-1* λ^- *F- HK022:*P_{sulA}*-mGFP P21:*P_{tet01}*-mKate2*	See Jaramillo-Riveri *et al.* (2022) for construction (CGSC 7740)
JKB22	MG1655	eSJR206 Δ*motA::Kn*^{R}	eSJR206 P1 using JW1879-2
JKB43	MG1655	eSJR206 Δ*motA*	JKB22 pE-FLP using pSJR017

Appendix Table S3: **List of plasmids.**

**Plasmid**	**Purpose**	**Source**
pSJR017	Clone-integration marker excision	pE-FLP (St-Pierre *et al.*, 2013)

Appendix Table S4: **List of primers.**

**Primer**	**5′-3′ Sequence**	**Purpose**
oJB01	`CTTTTGGCTTTGCGTCGTTTG`	*motA* deletion verification FW
oJB02	`TTCCCAGAATCCTGCCGATA`	*motA* deletion verification RV

2.2 Bulk experiments

2.2.1 Checkerboard assay

The inoculum was prepared as follows: An LB overnight culture (strain SJR206) was prepared as described in the Methods. Cells were then diluted (1:2000) into 5 mL of M9-based medium and incubated overnight at 37°C with agitation (150 rpm). In the morning, another 1:200 dilution was performed and the culture was incubated until $OD_{600} = 0.2$. In flat-bottomed 96-well plates (Corning Costar), a concentration series for each antibiotic was set up in all columns (drug A) or rows (drug B) as follows: Antibiotics were made up at double the highest concentration desired in 5 mL M9-based medium. A gradient dilution was set up on the x-axis by adding 100 μL of drug A to all wells in the first column and a serial dilution was performed for the remaining columns of wells up to column 10, with drug A not added to column 11 (Appendix Figure S2). Thus, each well was made up to a volume of 100 μL with double the desired drug concentration. A second drug gradient was similarly set up across the y-axis (rows) for drug B, again at double the desired concentration, with drug B not added to row H. The desired concentration was achieved in drug combination wells when the two antibiotic solutions are added (100 μL drug A + 100 μL drug B = 1:2 dilution). For wells administered with a single drug (column 11 and row H), 100 μL sterile medium was added to make up the final concentration (1:2 dilution). All wells were then mixed using a pipette. No drugs were added to column 12 which contained the positive and negative controls. At one edge of the plate (column 12), four wells were filled with 200 μL growth medium and inoculum (positive growth controls) and the other four with just 200 μL growth medium (negative controls for contamination). All wells of the plate (except the negative controls) were inoculated at a 1:1000 dilution using the prepared culture described above. All solutions were pre-warmed at 37°C.

A lid was taped onto the 96-well plate to minimise evaporation and debris formation (the tape prevents movement of the lid during shaking). The 96-well plate was then inserted into the FLUOstar Omega microplate reader (BMG LABTECH) and OD_{600} was measured for each well every 7 min for 28-50 hours depending on the growth medium. The platereader maintained a temperature at 37°C and shaking at 700 rpm using the double-orbital shaking mode. All optical densities (ODs) were blank corrected (subtracted by initial OD of sterile medium containing no antibiotic) and then exported to an Excel spreadsheet and imported to MATLAB for further analysis.

Growth curves from the platereader were noisy and sometimes contained extreme values considered to be measurement errors. Therefore, growth curves were smoothed with a 5-window moving median and outliers corrected using the *filloutliers* function in MATLAB. Outliers that were missed were manually corrected. Growth rates in each well were then measured via linear regression (*regress* function) to log-transformed OD_{600} from the exponential phase of the growth curve (between $0.025 < OD_{600} < 0.25$). Fits with an R^2 value below 0.8 were discarded. Growth rates were then normalised by the control growth rate (averaged from five wells). To reduce noise, a cubic smoothing spline was used for interpolation to the measured growth rates using the *csaps* MATLAB function. The dose response surface (linearly interpolated isoboles) was then plotted using the *contour* function in MATLAB.

2.2.2 Bulk growth rates

Population (bulk) growth rates were measured automatically using OGI-BIO bioreactors. The bioreactor was was calibrated according to the manufacturer instructions. The inoculum (strain JKB43) was prepared in LB as described previously in the Methods. The LB overnight culture was then sub-cultured at a 1:1000 dilution into 15 mL of M9-based medium and grown until OD=0.1-0.2 (37°C, 150 rpm). Glass reactor tubes containing 15 mL of M9-based medium were then inoculated at a 1:100 dilution using the prepared culture. Antibiotics were added at the appropriate concentration (CIP at 3 ng/mL and TET at 0.2 μg/mL) where necessary. Three independent replicates were performed for each of four conditions: No-drug control, CIP, TET, and CIP-TET. The bioreactor was incubated at 37°C for 24 hours. The cultures were mixed at 2000 rpm by a magnetic stir bar. OD measurements were taken every 5 minutes. The excel output file containing the OD measurements were then analysed in MATLAB. Growth rates were estimated via linear regression to the log-transformed growth curves during the exponential growth phase. Doubling rates (λ) were converted from growth rates (μ) as: $\lambda = ln(2) \times \mu$.

2.3 Mother machine

2.3.1 Microfluidic device dimensions

Appendix Table S5: **Mother machine microchannel dimensions used for different growth media**.

Growth Medium	Height (μm)	Width (μm)	Length (μm)
Gly	0.95	1.1	24
Glu	1.10	1.1	26
Glu-aa	1.10	1.2	26

2.3.2 Classification of cell fate

Using a simple algorithm, we classified cells into three categories by whether they continued growth and division ('survived'), abruptly stopped growth and division ('died'), or excessively filamented beyond the length of the microchannels (Appendix Figure S9). We quantified the fate of mother cell lineages over the duration of the experiment (24 hours), which included a 10 hour recovery period. This ensures that cells initially classified as 'dead' were not persister cells (i.e. resumed growth post antibiotic treatment). First, lineages that excessively filamented (length <20 μm) were separated and classified as 'hyper-filamented'. These cells extended beyond the length of the microfluidic microchannels (cell traps). The remaining lineages were classified as 'died' if their last recorded elongation rate is below a threshold value, or if they did not divide for 4 hours or more. The elongation rate threshold was set at 20% of the median elongation rate of the no-drug control condition for each growth medium. In some rare cases cells lysed – immediately losing their physical integrity – and could no longer be visually tracked, therefore escaping the filters set above. These cells were visually identified and manually classified as 'died'.

The majority of cells that ceased growth maintained physical integrity and did not resume growth after removal of antibiotic treatment. These cells exhibited, after a delay, an exponential decline in mKate2 intensity (constitutive gene expression) once growth had stopped and did not recover (Appendix Figures S4-S6F, L). Given that elongation (dilution) had ceased, this decline indicates that constitutive expression and metabolic activity/protein production has stopped completely. The exponential decline in mKate2 intensity is likely due to photobleaching of the fluorescent protein. This observation further suggests that these cells are most likely dead or in the process of dying. We can therefore use the constitutive expression trajectories to verify the classification of dead cells. An example of mother cell lineages classified by fate is shown in Appendix Figures S4-S6. There was good separation between 'survived' and 'dead' lineages based on the elongation rates and constitutive gene expression trajectories.

2.3.3 Quantification of cell survival and death

Survival analysis was carried out using the the Kaplan-Meier (K-M) estimator (Kaplan & Meier, 1958), a non-parametric model commonly used for survival analysis. First, we organised our data into two columns, with the first column populated with lineage lifespans (survival times) in hours, and the second column denoting whether a death event occurred or not (Boolean, true or false). Not all cells died

during the experiment – these are known as right-censored individuals and are important to include in our statistic to avoid overestimating the rate of death. Lifespans were determined from the start of the experiment until the time of death or time of censorship, where censorship refers to the end of the experiment or until lineage tracking is lost. For lineages classified as 'dead' (see above), the time of death was determined as the start of the cell-cycle during which cell death occurred (i.e. time of birth of the dead cell). Each lineage is made up of a succession of cell-cycles. Here, a cell-cycle represents the period between birth and division or between birth and the last recorded image. A dead cell therefore represents a lineage's final cell-cycle. Although we could not precisely determine the fate of lineages that escaped due to filamentation, we lumped them with dead lineages in the survival curves since they are not growing 'normally' and to avoid bias if these were removed from the calculation. The survival function S(t) was estimated using the K-M model (Kaplan & Meier, 1958), defined as:

$$\hat{S}(t) = \prod_{t_i<t} \frac{n_i - d_i}{n_i} \tag{1}$$

where d_i are the number of death events at time t and n_i is the number of subjects at risk of death just prior to time t. The K-M estimate represents an accumulation of probabilities equivalent to *S(t)* = $\mathbb{P}$(surviving past time t). The analysis was implemented using the *lifelines* package in Python (Davidson-Pilon, 2019).

2.3.4 Classification of dead lineages into low-SOS and high-SOS inducers

The cells that died during the experiment (fate classification described above) were split into two groups based on their SOS expression. The "high inducer" cells undergo a jump in SOS expression correlated to the moment they stop elongating (for example see Appendix Figure S5D & E) while "low inducers" stop expressing SOS and their fluorescence simply decay (both groups can be seen in Appendix Figure S11D-F). To detect such groups we applied a threshold on the maximum SOS value of each trajectory, since high inducers present a fluorescence peak. Trajectories whose maximum value are above the threshold were classified as high inducers while other trajectories were classified as low inducers (see the two populations in orange and magenta respectively in Appendix Figures S11-S12). This value was set independently for each condition and replicate. A density plot of the maximum values of each trajectory often show two distinct distributions for the high and low SOS inducers (see Appendix Figure S12) and the threshold value was set to the valley between those populations when possible, with additional manual adjustments when needed. The list of thresholds for each condition and replicate are shown in Appendix Table S6.

Appendix Table S6: **Threshold log values used for classification of low and high-SOS inducers**. Values for different replicates separated with commas.

	Control	CIP	TET	CIP-TET
Gly	7.09,7.35,7.48	7.6,7.56	6.59,6.68,7.28	7.06,7.14
Glu	7.2,7.2	7.7,7.96	7.08,7.10	6.93,6.85
Glu-aa	7.04,7.01	7.6,7.37,7.29	7.31,6.94	7.19,7.14

2.4 Statistical analysis of Bliss independence

Description of statistical model below adapted from Demidenko & Miller (2019).

According to Bliss (Bliss, 1939), drugs act independently if the survival fraction (*Sf*) of cells upon simultaneous treatment (Sf_{AB}) is equal to the product of *Sfs* of cells (Sf_A and Sf_B) under single-drug treatment:

$$Sf_{AB} = Sf_A \times Sf_B, \tag{2}$$

or equivalently as the log-transformed sum:

$$\ln Sf_{AB} = \ln Sf_A + \ln Sf_B. \tag{3}$$

Let there be n_1 replicate experiments in drug group A, n_2 replicates in drug group B, and n_3 replicates in drug group D when drugs with the single dose from groups A and B are given simultaneously. Since the observed *Sfs* are positive we can model the variation of *Sf* on the log scale, which can be expressed through the exponential function as follows:

$$A_j = e^{\mu_1+\epsilon_{1j}}, \quad B_j = e^{\mu_2+\epsilon_{2j}}, \quad D_j = e^{\mu_3+\epsilon_{3j}}, \tag{4}$$

where ϵ_{1j}, ϵ_{2j}, ϵ_{3j} denote normally distributed unobserved errors with zero mean and common variance σ^2. The true unknown μs are expected to be negative and connected to unknown true surviving fractions from 3 as $Sf_A = e^{\mu_1}$, $Sf_B = e^{\mu_2}$ and $Sf_{AB} = e^{\mu_3}$. We will test the hypothesis that

$$\ln Sf_A + \ln Sf_B - \ln Sf_{AB} = 0 \tag{5}$$

by statistical means using replicates in each group. Let the log fractions in each group be $y_{1i} = \ln A_i$, $y_{2i} = \ln B_i$ and $y_{3i} = \ln D_i$. Then the system 4 can be rewritten as an ANOVA model with three groups,

$$y_{ij} = \mu_i + \epsilon_{ij}, \quad i = 1, 2, 3, \quad j = 1, ..., n_i \tag{6}$$

where n_i in the number of replicates in the ith group. Now the drugs independence can be conventionally expressed in terms of μs as the linear null hypothesis:

$$H_0 : \mu_1 + \mu_2 - \mu_3 = 0 \tag{7}$$

This hypothesis is tested using the test statistic T which has a t-distribution with $\sum_{i=1}^{3} n_i - 3$ degrees of freedom, namely,

$$T = \frac{(\bar{y}_1 + \bar{y}_2 - \bar{y}_3)\sqrt{\sum_{i=1}^{3} n_i - 3}}{\sqrt{\sum_{i=1}^{3}\sum_{j=1}^{n_i}(y_{ij} - \bar{y}_i)^2 \sum_{i=1}^{3} n_i^{-1}}} \tag{8}$$

where $\bar{y}_1$, $\bar{y}_2$ and $\bar{y}_3$ are the average log fractions in groups A, B and D. To test the null hypothesis of drugs independence, we compute the two-sided the p-value; to test for synergy or antagonism we

compute the one-sided p-value.

References

Baba T, Ara T, Hasegawa M, Takai Y, Okumura Y, Baba M, Datsenko KA, Tomita M, Wanner BL, Mori H (2006) Construction of Escherichia coli K-12 in-frame, single-gene knockout mutants: the Keio collection. *Mol Syst Biol* **2**: 2006.0008

Bliss CI (1939) The toxicity of poisons applied jointly. *Ann Appl Biol* **26**: 585–615

Davidson-Pilon C (2019) lifelines: survival analysis in Python. *J Open Source Softw* **4**: 1317

Demidenko E, Miller TW (2019) Statistical determination of synergy based on Bliss definition of drugs independence. *Plos One* **14**: e0224137

Jaramillo-Riveri S, Broughton J, McVey A, Pilizota T, Scott M, El Karoui M (2022) Growth-dependent heterogeneity in the DNA damage response in Escherichia coli. *Mol Syst Biol* **18**: e10441

Kaplan EL, Meier P (1958) Nonparametric Estimation from Incomplete Observations. *J Am Stat Assoc* **53**: 457

Loewe S (1928) Die quantitativen Probleme der Pharmakologie. *Ergebnisse der Physiologie* **27**: 47–187

Loewe S (1953) The problem of synergism and antagonism of combined drugs. *Arzneimittel-Forschung* **3**: 285–290

St-Pierre F, Cui L, Priest DG, Endy D, Dodd IB, Shearwin KE (2013) One-step cloning and chromosomal integration of DNA. *ACS Synth Biol* **2**: 537–541
