# Supplementary material for: Suppression of bacterial cell death underlies the antagonistic interaction between ciprofloxacin and tetracycline
Source: Mol Syst Biol. 2025 Oct 27;22(1):7. doi: 10.1038/s44320-025-00162-w (PMC12759072; doi:10.1038/s44320-025-00162-w)
Supplement: Supplementary file 5 — Expanded View Figures [file 44320_2025_162_MOESM5_ESM.pdf]

Expanded View Figures

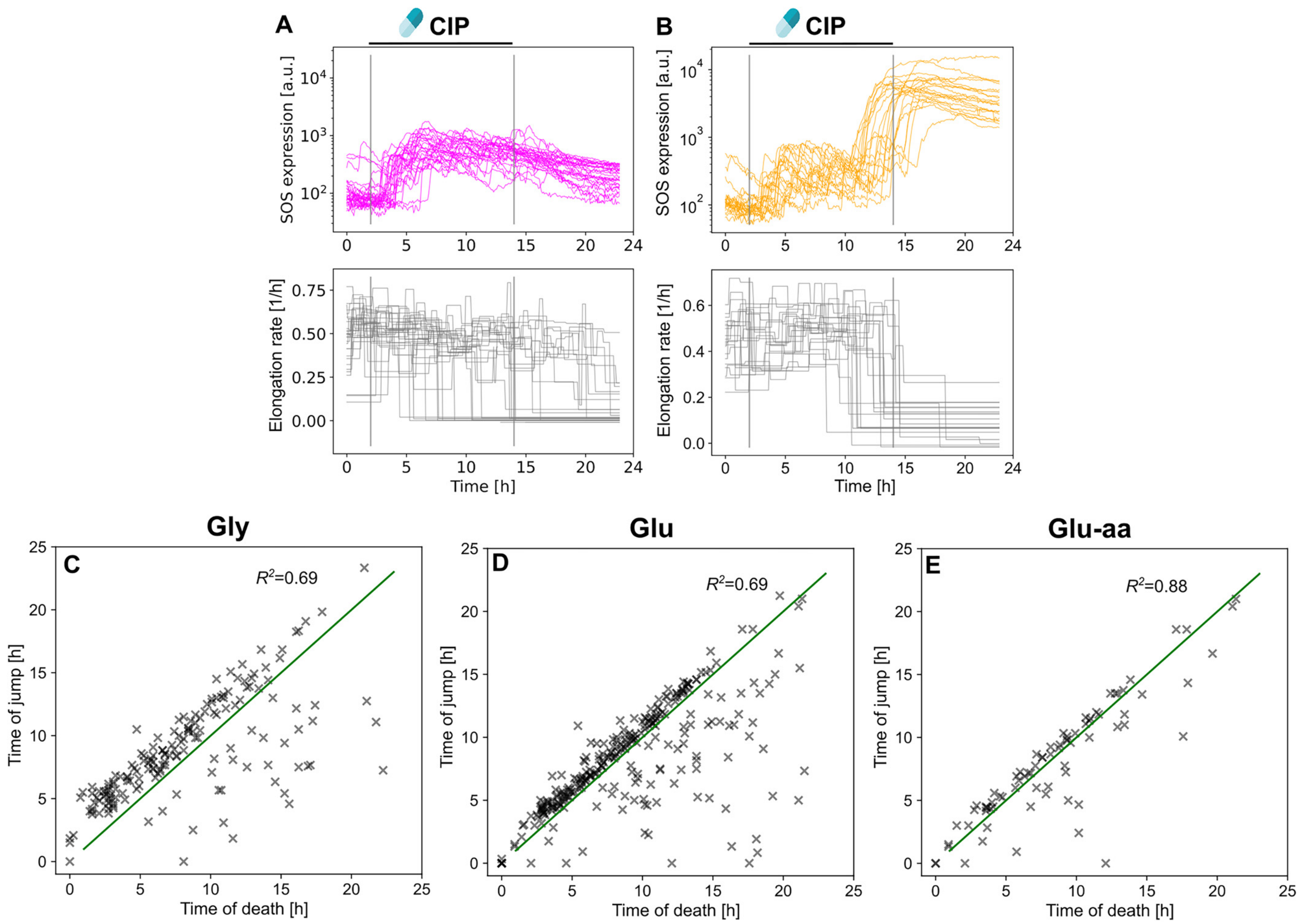

Figure EV1. High-SOS stress jump correlates with the time of elongation arrest.

(**A**) Single-cell trajectories of SOS expression and elongation rate for low-SOS cells that died under CIP treatment for growth in the Glu medium. (**B**) Single-cell trajectories of SOS expression and elongation rate for a representative group of high-SOS cells that died at similar times under CIP treatment for growth in the glu medium. We noticed that elongation arrest correlated with the time of jump in SOS levels. One group of cells with similar times of death from one experiment were identified and plotted in (**B**). CIP was introduced between hours 2–14 (vertical lines). (**C–E**) Time of death (defined by the time of elongation arrest) plotted against the time of jump (determined by the time the SOS expression crosses 1300 a.u.) for individual high-SOS cells (black crosses) from CIP treatment experiments. The green line shows the x = y function and R^2 is the correlation coefficient.

 © The Author(s)

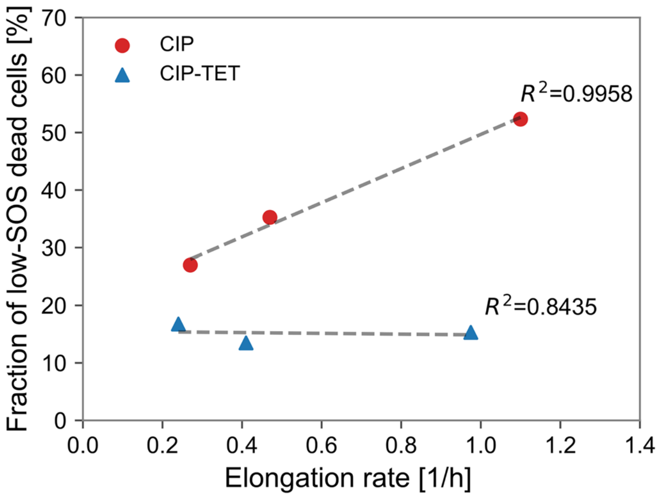

Figure EV2. Tetracycline eliminates growth-dependence in low-SOS dead cells under ciprofloxacin.

Fraction of low-SOS cells that died under CIP and CIP-TET treatment in different growth conditions. Fractions are plotted against the median single-cell elongation rate of surviving cells calculated from the final 2 h of antibiotic treatment. R^2 is the correlation coefficient.
